# Supplementary material for: Exploring experiences engaging in exercise from the perspectives of women living with HIV: A qualitative study
Source: PLoS One. 2023 Jun 2;18(6):e0286542. doi: 10.1371/journal.pone.0286542 (PMC10237415; doi:10.1371/journal.pone.0286542)
Supplement: S1 File — (PDF) [file pone.0286542.s001.pdf]

S1 File: Research Ethics Board Approval Letter

OFFICE OF THE VICE PRESIDENT,
RESEARCH AND INNOVATION

RIS Protocol Number: 40852

Approval Date: 3-May-21

PI Name: Miss Kelly O'Brien

Division Name:

Dear Miss Kelly O'Brien:

Re: Your research protocol application entitled, "Exploring experiences engaging in exercise from the perspectives of women living with HIV: A qualitative study"

The HIV REB has conducted a Delegated review of your application and has granted approval to the attached protocol for the period 2021-05-03 to 2022-05-02.

If this research involves face-to-face (F2F) in person research, please note that REB approval alone is not sufficient to commence research. You must wait for an approval letter from the F2F COVID-19 Review Committee. The approval letter will be sent to the Principal Investigator's email address once the Committee has deemed the F2F in-person research ready to start.

Please be reminded of the following points:

- An **Amendment** must be submitted to the REB for any proposed changes to the approved protocol. The amended protocol must be reviewed and approved by the REB prior to implementation of the changes.
- An annual **Renewal** must be submitted for ongoing research. Renewals should be submitted between 15 and 30 days prior to the current expiry date.
- A **Protocol Deviation Report** (PDR) should be submitted when there is any departure from the REB-approved ethics review application form that has occurred without prior approval from the REB (e.g., changes to the study procedures, consent process, data protection measures). The submission of this form does not necessarily indicate wrong-doing; however follow-up procedures may be required.
- An **Adverse Events Report (AER)** must be submitted when adverse or unanticipated events occur to participants in the course of the research process.
- A **Protocol Completion Report** (PCR) is required when research using the protocol has been completed. For ongoing research, a PCR on the protocol will be required after 7 years, (Original and 6 Renewals). A continuation of work beyond 7 years will require the creation of a new protocol.
- If your research is funded by a third party, please contact the assigned Research Funding Officer in Research Services to ensure that your funds are released.

Best wishes for the successful completion of your research.

		Protocol #:26982			
Status:Delegated Review App	Version:0002	Sub Version:0000	Approved On:3-May-21	Expires On:2-May-22	Page 13 of 13

OFFICE OF RESEARCH ETHICS
McMurrich Building, 12 Queen's Park Crescent West, 2nd Floor, Toronto, ON M5S 1S8 Canada
Tel: +1 416 946-3273 ● Fax: +1 416 946-5763 ● ethics.review@utoronto.ca ● http://www.research.utoronto.ca/for-researchers-administrators/ethics
