# Supplementary material for: Exploring experiences engaging in exercise from the perspectives of women living with HIV: A qualitative study
Source: PLoS One. 2023 Jun 2;18(6):e0286542. doi: 10.1371/journal.pone.0286542 (PMC10237415; doi:10.1371/journal.pone.0286542)
Supplement: S2 File — (PDF) [file pone.0286542.s002.pdf]

S2 File: Interview Guide

Thank you for agreeing to participate in this study. I am meeting with you to understand your experiences engaging in exercise. Despite the benefits of exercise, physical activity levels among people living with HIV remain significantly low. More specifically, gender significantly influences exercise patterns, with women living with HIV engaging in lower exercise compared to their male counterparts. As such, I am interested in learning about your level of engagement with exercise, what are your motivations and/or challenges to exercising, and to obtain any recommendations you may have for facilitating the uptake of exercise. The findings from the interview may be used to help better understand exercise among women living with HIV and better tailor exercise programs with women living with HIV.

Before we continue with the interview, it is also important to clearly define what is meant by physical activity and exercise. **Physical activity** is defined as general movement that occurs on a day-to-day basis, including walking your dog or gardening. **Exercise** is defined as structured and planned movement that is completed with the intention of maintaining overall health, including performing 15 minutes of strengthening exercises 5 times a week. For the purpose of this study, I am interested in your experiences with exercise, however, I will also be asking you questions to inquire further about your thoughts around exercise vs. physical activity- including its terminology, perspectives on, differences, and preferences.

You may decide to not answer questions that you do not want to, and you may end the interview at any time. I will also be recording this interview using a separate audio-recorder and taking notes to capture some important thoughts or opinions that are brought up during our discussion. After the interview, you will be asked to complete a demographic questionnaire.
Do you have any questions before we begin?

Start recorder after all questions addressed

OBJECTIVE 1: to understand the nature and extent women living with HIV engage in exercise

1) What experience with exercise, if any, have you had up until now?
Potential Probes:
Can you please expand on that?

2) How often do you engage (or take part) in exercise?
Potential Probes:
What types of exercise activities do you/have you engage(d) with?
Where do you exercise?/Where have you exercised in the past?
How often?
How long do you exercise per week?
Are there certain activities you prefer? Why?
Do you exercise alone or with other people?

<u>For those who exercise:</u>

4) What are your reasons for exercising?
Potential Probes:
Can you tell me what are you motivating factors for wanting to exercise?

5) What are some positive aspects of your experiences with exercise?
Potential Probes:
Can you tell me more about that […]?
Would you like to exercise more? (i.e., hours per week?) If so, why?

6) What are some negative aspects of your experience with exercise?
Potential Probes:
Can you tell me more about that […]?
Would you like to exercise more? (i.e., hours per week?) If so, why?

7) Has the COVID-19 Pandemic affected your taking part in exercise?
Potential Probes:
How so? Increased? Decreased? Made it easier or more difficult?
Tell me more about that.
What has your exercise been like during the pandemic?
Has this been going on from the beginning of the pandemic?
Have you tried doing at-home exercise/activities?
Have you tried taking part in any online (web-based) forms of exercise activity?

<u>For those who do NOT exercise:</u>

8) What are your reasons for not exercising?
Potential Probes:
Can you tell me more?

9) Would you like to exercise more? (i.e., hours per week?) If so, why?
Potential Probes:
Can you tell me more about that […]?
What do you think could help you to exercise? i.e., family support, financial support…etc.

OBJECTIVE 3: to identify the facilitators and barriers (factors that influence) to engaging in exercise

<u>For those who exercise:</u>

10) Have you faced any **challenges** when trying to engage in exercise?
Potential Probes:
Tell me more about that
What factors contribute to your participation and/or lack of participation with exercise?
How do *personal factors* impact whether you engage in exercise?

How do *environmental factors* impact whether you engage in exercise?
Would you be willing to take part in an online exercise program if available?

11) What **motivates** you to exercise?
Potential Probes:
Why? How so?
Has COVID-19 affected this?

12) Do you think your **gender** has an influence on your level of engagement with exercise? How so?
Potential Probes:
Can you tell me more about that […]?

13) Existing research suggests that women living with HIV can face more barriers when trying to engage in exercise compared to men, including: social and cultural expectations (i.e., child-bearing and housecleaning work), economic limitations, and lack of support. What are your thoughts on this?
Potential Probes:
Have any of these factors influenced you?
Can you relate to such barriers? Please explain.

<u>For those who do NOT exercise:</u>

14) Have you faced any **challenges** when trying to engage in exercise?
Potential Probes:
Tell me more about that
What factors contribute to your participation and/or lack of participation with exercise?
How do *personal factors* impact whether you engage in exercise?
How do *environmental factors* impact whether you engage in exercise?
Would you be willing to take part in an online exercise program if available?

15) What could potentially **motivate** you to exercise?
Potential Probes:
Why? How so?
Has COVID-19 affected this?

16) What **discourages** you from exercising?
Potential Probes:
Why? How so?
Has COVID-19 affected this?

17) Do you think your **gender** has an influence on your level of engagement with exercise? How so?
Potential Probes:
Can you tell me more about that […]?

18) Existing research suggests that women living with HIV can face more barriers when trying to engage in exercise compared to men, including: social and cultural expectations (i.e., child-bearing and housecleaning work), economic limitations, and lack of support. What are your thoughts on this?

Potential Probes:

Have any of these factors influenced you?
Can you relate to such barriers? Please explain.

OBJECTIVE 2: to understand the perceived impact of engaging in exercise

19) What is your general attitude towards exercise?

Potential Probes:

Do you think exercise has a positive impact on you? How so?
How do you think exercise impacts your health and overall quality of life?

20) In your opinion, how important is exercise for the health of adults living with HIV?

Potential Probes:

How might exercise help with HIV, aging and multi-morbidity?

21) To what extent do you think exercising will help improve your health outcomes living with HIV?

Potential Probes:

Can you tell me more about that?
Can you give me some examples of how?

22) Are you currently satisfied with how much you exercise?

Potential Probes

Do you want to engage with it more?
Will this help you in other aspects of your life?
Why?

OBJECTIVE 4: to identify strategies and recommendations for facilitating uptake and sustainability of exercise

23) How important do you think it is to promote exercise among women living with HIV?

Potential Probes:

Why?

24) How do you think you could incorporate more exercise into your life?

Potential Probes:

In what ways can you/do you mitigate the challenges to engaging in exercise?

25) The term ‘physical activity’ is used sometimes interchangeably with ‘exercise.’ What are your thoughts on the difference between the terminology?

Potential Probes:

Do you think the terms means different things? Is so, describe them.

What do you think the difference in exercise and physical activity are?
Does it cause you to become confused? How so?

26) Do you think knowing the terms makes a difference when recommending exercise among women living with HIV?
Potential Probes:
Tell me more, please.

27) Do you consider yourself physically active?
Potential Probes:
Please expand on this.

28) Overall, what recommendations might you have for ways in which to help facilitate exercise among women with HIV?

29) Would having someone else exercise with you encourage you to exercise regularly?
Potential Probes:
Why?

30) How would you like to see more people living with HIV, specifically women, access exercise?
Potential Probes:
How would you access exercise activities?
What kinds of resources do you think can encourage you or other women living with HIV to engage in more exercise?

Before we finish this interview, would you like to say anything about your experience with exercise that was not discussed? Thank you for taking the time to participate in this interview. The information you have provided me will help me gain a better understanding of your experiences engaging in exercise. If you have any questions or concerns regarding this research, please contact me via email (nora.sahel.gozin@mail.utorotono.ca) or by phone at 416-946-3935. My contact information is also listed on the consent form. Again, thank you for your time and cooperation. It is greatly appreciated.

Now that we are done with the interview, I am going to ask you a few questions about yourself in the demographic questionnaire.
