# Supplementary material for: Exploring experiences engaging in exercise from the perspectives of women living with HIV: A qualitative study
Source: PLoS One. 2023 Jun 2;18(6):e0286542. doi: 10.1371/journal.pone.0286542 (PMC10237415; doi:10.1371/journal.pone.0286542)
Supplement: S3 File — (PDF) [file pone.0286542.s003.pdf]

S3 File: Details of Exercise Engagement (type, frequency and duration) among women living with HIV based on interview data

Participant Number	Type	Frequency	Duration	Classification*
**1**	○ Walking	○ Infrequently		Non-exerciser
**2**	○ Walking	○ 2 times/week	○ One hour/week	Non-exerciser
**3**	○ Walking<br>○ Calisthenics<br>○ Kick boxing<br>○ Yoga trapeze, resistance bands, weights, yoga<br>○ Isolating and resistance exercises<br>○ Cycling in summer	○ Walks everyday<br>○ Exercises everyday	○ Walking: 2 hours/day<br>○ Exercises: 1 hour/day	Exerciser
**4**	○ Group-based exercise class (including mindfulness)	○ Once a week	○ One hour/week	Non-exerciser
**5**	○ Zumba<br>○ Climbing staircase<br>○ Brisk walking	○ Staircase climbing: 5 days/week<br>○ Brisk walking: 5 days/week<br>○ Occasional Zumba	○ Walking and climbing: ~ 2 hours (8 km/day)<br>○ Zumba ~ 1 hour	Exerciser
**6**	○ Walking/jogging, cycling<br>○ Weights<br>○ Yoga group-based exercise (online)	○ Walking/jogging, cycling, weights: spontaneous (unknown)<br>○ Yoga: 1x/week	○ Yoga: 30 minutes/week	Non- Exerciser
**7**	○ Cycling<br>○ Cardio group-based exercise class	○ Cycling: 2x/week<br>○ Cardio: 5x/week	○ Cycling: unknown<br>○ Cardio: 5 hours/week	Exerciser
**8**	○ Has not been active for past 6 years			Non-Exerciser
**9**	○ Hiking/walking (stopped spring 2021)	○ 1x/week	○ 2.5 hours/week	Non-Exerciser
**10**	○ Cycling (stopped 2.5 weeks before interview)	○ 7x/week	○ 7 hours/week	Non-Exerciser

LEGEND: *We defined 'engagement in exercise' using the Canadian Society of Exercise Physiology (CSEP) physical activity guidelines as currently accruing (at the time of the interview) at least 150 minutes of moderate-to-vigorous-intensity aerobic physical activity per week.
